# MGFD: the maize gene families database

**DOI:** 10.1093/database/baw004

**Published:** 2016-02-20

**Authors:** Lei Sheng, Haiyang Jiang, Hanwei Yan, Xiaoyu Li, Yongxiang Lin, Hui Ye, Beijiu Cheng

**Affiliations:** Key Laboratory of Crop Biology of Anhui Province, Anhui Agricultural University, Hefei 230036, China

## Abstract

Most gene families are transcription factor (TF) families, which have fundamental roles in almost all biological processes (development, growth and response to environmental factors) and have been employed to manipulate various types of metabolic, developmental and stress response pathways in plants. Maize (*Zea mays*) is one of the most important cereal crops in the world due its importance to human nutrition and health. Thus, identifying and annotating all the gene families in maize is an important primary step in defining their functions and understanding their roles in the regulation of diverse biological processes. In this study, we identified 96 predicted maize gene families and systematically characterized all 5826 of the genes in those families. We have also developed a comprehensive database of maize gene families (the MGFD). To further explore the functions of these gene families, we extensively annotated the genes, including such basic information as protein sequence features, gene structure, Gene Ontology classifications, phylogenetic relationships and expression profiles. The MGFD has a user-friendly web interface with multiple browse and search functions, as well as data downloading. The MGFD is freely available to users at http://mgfd.ahau.edu.cn/.

**Database URL**: http://mgfd.ahau.edu.cn/

## Introduction

A gene family is a set of several similar genes, formed by duplication of a single original gene and generally with similar biochemical functions. These genes encode instructions for making products (such as proteins) that have a similar structure or function. Classifying individual genes into families helps researchers describe how genes are related to each other. The genes in the same family can closely packed together to form a gene cluster, but most of the time, they are scattered in different locations in the same chromosome or exist in different chromosomes. Researchers can use gene families to predict the function of newly identified genes based on their similarity to known genes.

Maize (*Zea mays*) is an important cereal crop that has also become an important model species for the study of genetics, evolution, and other basic biological processes in plants. Many of the characterized maize gene families consist of important transcription factors (TFs), such as heat shock transcription factor (hsf) (1), MADS-box (2), and WRKY gene families (3). Transcription factors are the key regulators of gene expression and play critical roles in the life cycles of higher plants (4). TF families in plants are well characterized, and several databases for plant TFs have been developed (5–7). However, until now, there is not a comprehensive list of gene families or a database characterizing all the gene families in the maize genome. Given the importance of maize gene families, there is a strong need for a database that integrates multiple sources of information to give a comprehensive, genome-wide view of gene families in maize.

With this in mind, we assembled a comprehensive list of maize gene families through manual reviews of the literature. We then predicted genes for all of these families in the maize genome and constructed a comprehensive database that we call the Maize Gene Families Database (MGFD) (http://mgfd.ahau.edu.cn/). In particular, the MGFD provides comprehensive information for individual genes as well as many other annotations of the maize gene families. The database has a user-friendly interface that can be used to display and search the detailed annotations. It is our objective that the MGFD will become a useful resource for the plant genetics research community, especially in the areas of bioinformatics and genomics.

## Identification of maize gene families

We combined automated search and manual confirmation to generate a collection of maize gene families that is as complete as possible according to The Arabidopsis Information Resource (TAIR) (https://www.arabidopsis.org/), which contains gene structure, gene product information, gene expression, genome maps and information about the *Arabidopsis* research community (8). Maize genome sequences were downloaded from http://www.maizesequence.org/(Release 22).

At the very start, we searched the domains of each gene family in *Arabidopsis* by means of The Arabidopsis Information Resource (TAIR) (https://www.arabidopsis.org/). Then the Hidden Markov Model (HMM) profile of the domains were employed as a query to identify all possible genes in the maize genome using the BlastP program (*P*  =  0.001). Therefore, we named maize gene families with reference to the terminology of gene family in *Arabidopsis*. In order to identify the maximum number of these domain-containing sequences, two different HMM profiles were adopted in the gene searches. The first was obtained from the Pfam database (http://Pfam.sanger.ac.uk/Software/Pfam) (9), and the second profile was generated by alignments to genes in *Arabidopsis* (10). Second, the Pfam database was used to determine whether each of the candidate sequences was a member of its gene family. To exclude overlapping genes, all of the candidate genes were aligned using ClustalW (11) and checked manually. Finally, we identified 5826 genes in maize and organized them into 96 gene families.

## Analysis and annotation of maize gene families

To provide comprehensive information for the identified gene families, we made extensive annotations at both the family and gene levels. For each gene family, a brief introduction is given on the family page. The physical locations, coding strand and protein lengths were obtained from Phytozome, and the calculated isoelectric points (PI) and molecular weights (Mw) were obtained from Expasy (12) (http://www.expasy.org/). The phylogenetic trees were generated using MEGA v4.0 (13) with the neighbor-joining (NJ) method using the complete predicted protein sequences for the genes in each family. The complete amino acid sequences of each gene family were subjected to Multiple Expectation Maximization for Motif Elicitation (MEME) (14) analysis online (http://meme. sdsc.edu/meme4_3_0/intro.html). MapInspect software was then used to obtain location information for the maize gene families, and the publicly available transcriptome data (15) for maize was used to perform comprehensive expression analyses for all of the gene families, as well as all of the individual genes. The intron-exon organizations for the genes in each family were obtained from GSDS (http://gsds.cbi.pku.edu.cn/).

## Implementation and web interface

A web-based platform, the MGFD combines the MySQL (version 5.5.8) database management system with a dynamic web interface based on asp.net (version 4.0) and sqlservers2005.

**Figure 1:**
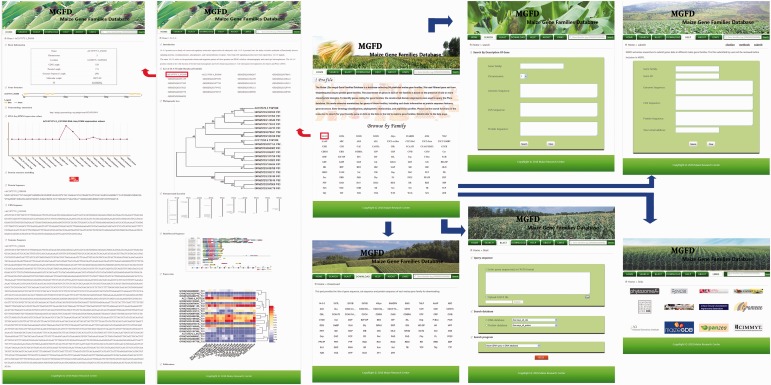
An illustration of the MGFD system.

The web interface of the MGFD was designed to comprise the following seven components: Home, Search, BLAST, Download, Help, About and Links. An illustration of the MGFD system is shown in Figure 1. MGFD has a user-friendly entry point for each gene family. We kept the database interface of 96 predicted gene families in maize. A uniform text query interface for each gene family was designed. Users can click on the name of each gene family to activate the annotation information page with detailed annotations (Figure 1). A page providing general information that includes an introduction, a list of family member genes, a phylogenetic tree, chromosomal distribution, motif-based sequence analysis and gene expression is shown. Furthermore, users can click on each gene to browse details, such as chromosome strand, physical location, PI, Mw, CDS length, protein length, genome sequence length, gene structure, etc.

The MGFD provides two different ways to search the data; a quick search and an advanced search. Users can either type a truncated version or the entire Gene ID (e.g., GRMZM2G010433) into the search field found at the top right of each page. In addition, an advanced search which includes gene family, chromosome, genome sequence, CDS sequence and protein sequence is constructed for users. Finally, users are able to easily navigate from their search results to pages containing detailed annotations. Moreover, BLAST search against all the maize genes is provided. All of the sequence information is available through the download page.

## Discussion

The goal of the MGFD is to be comprehensive in both the collection of maize gene families and the information provided for each gene family. The database consists of 96 predicted maize gene families with extensive annotations for genes in these families. Users can apply various kinds of information from our database based on their own needs and requirements.

We anticipate that the MGFD database will become a useful resource for the research community, and particularly for studies about the relationships between genes and gene families. We provide some comparisons demonstrating the utility of the database as follows.

(1) MGFD is a more comprehensive and professional database of maize gene families. At present, there are several databases for animal and plant TFs; examples are DATF for *Arabidopsis* (5), TFdb for mouse (16), FlyTF for Drosophila (17), AnimalTFDB for animals (18) etc. These databases focus only on TFs, while the MGFD database not only contains TF gene families, but also many other maize gene families. Our gene family number is the most and the most comprehensive in same kind databases.

(2) There is another database—ProFITS database (http://bioinfo.cau.edu.cn/ProFITS/), is also a more comprehensive database for corn gene family. Compared to the ProFITS database, the MGFD database has more powerful data and function. At first, the MGFD contains 96 maize gene families, while the ProFITS contains 58 TF families. In terms of gene number, the MGFD contains 5826 maize genes, while the ProFITS contains 2543 maize genes. Hence the amount of our database is much larger than that of ProFITS at both the family and gene levels. Secondly, we have made detailed bioinformatics analysis for each gene family, such as phylogenetic analysis, chromosomal distribution, motif-based sequence analysis and gene expression, while the ProFITS did not do any analysis for each gene family. Thirdly, we have made detailed bioinformatics analysis for each gene, such as chromosome strand, physical location, PI, Mw, CDS length, protein length, genome sequence length, gene structure, etc. Therefore, the MGFD database, by contrast, has more comprehensive information about maize genes. At last, the MGFD includes the Blast section, which will benefit users’ requirements.

(3) Compared to Gramene and Phytozome, the MGFD database is aiming at becoming a comprehensive database of maize gene families with extensive annotations for genes in these families.

Gramene (http://www.gramene.org/) (19) is a curated, open-source, integrated data resource for comparative functional genomics in crops and model plant species. Though it contains genetic and physical maps with genes, ESTs and QTLs locations, genetic diversity data sets, etc, it does not include any information about gene families. Therefore, compared to Gramene, the MGFD database concentrates on maize genes and gene families. The goal of the MGFD is to be comprehensive in both the collection of maize gene families and the information provided for each gene family, while the goal of Gramene is to facilitate the study of cross-species comparisons using information generated from projects supported by public funds.

Phytozome (http://phyto160zome.jgi.doe.gov/pz/portal.html) is the Plant Comparative Genomics portal that provides access to 61 sequenced and annotated green plant genomes, 47 of which have been clustered into gene families at 12 evolutionarily significant nodes. Compared to Phytozome, the MGFD database is much more direct for propaedeutic researchers who want to study maize genes and gene families. Moreover, the MGFD contains heat map of each gene family and RNA-Seq FPKM expression value of each gene, which makes our site more convenient to our users.

In addition, the MGFD database has a data submitting system that will enhance the utility of our database. One of the goals for the MGFD database is to provide the largest platform for the sharing of information about maize gene families across the world. With the development of high-throughput sequencing technologies, researchers will explore more biological data, such as the re-sequencing data, transcriptome data, the proteomic data, GWAS data, etc. Researchers who want to submit related data about maize genes and gene families may upload the files by selecting the ‘Submit’ button from the ‘Help’ page.

Therefore, maize researchers will benefit from using the MGFD because in a single reference, they have access to the broadest compendium of maize gene families available. We expect that the MGFD database will be an extremely valuable resource and strive to make our site better and more user friendly for the research community.

## Conclusions

MGFD is a comprehensive database of maize gene families with extensive annotations for genes in these families, including basic information, protein sequence features, gene structure, Gene Ontology, transcriptome data, etc. Because we have established an operational pipeline for maize gene family identification and annotation, it will be relatively straightforward for us to update the database regularly as more maize gene data becomes available. In the coming years, we plan to add more gene annotations and biological data to enrich our database, as well as to incorporate more information from the research community into our database to better serve the users.
